# Testing the waters on home-based albuminuria screening to enhance the detection and management of CKD

**DOI:** 10.1093/ckj/sfae031

**Published:** 2024-02-09

**Authors:** Lauren Floyd, Pierre Delanaye

**Affiliations:** Renal Department, Royal Preston Hospital, Lancashire Teaching Hospitals, Lancashire, UK; Division of Cardiovascular Sciences, The University of Manchester, Manchester, UK; Department of Nephrology-Dialysis-Transplantation, University of Liège, CHU Sart Tilman, Liège, Belgium; Department of Nephrology-Dialysis-Apheresis, Hôpital Universitaire Carémeau, Nîmes, France

Chronic kidney disease (CKD) is one of the leading causes of morbidity and mortality globally. In recent years, advances in treatment strategies have shown great promise to reduce the rate of progression and better manage patients with CKD. This has been largely driven by understanding the role of proteinuria and albuminuria in the loss of kidney function. Proteinuria denotes an increased presence of protein in the urine, while albuminuria is characterized by an abnormal loss of albumin in the urine [1]. In normal adults, urinary protein excretion is typically less than 200 mg/day, with albumin excretion below 30 mg/day. Both proteinuria and albuminuria serve as independent risk factors for progressive CKD and cardiovascular disease (CVD). Notably, the Kidney Disease: Improving Global Outcomes (KDIGO) guidelines advocates for screening and monitoring of albuminuria over proteinuria, as it is more sensitive in detecting glomerular injuries and an easier-to-standardize measurement. Albuminuria is therefore considered the gold standard for quantifying urine protein in most kidney diseases [1]. While 24-hour urine collections have previously been required, the urine albumin creatinine ratio (ACR) correlates well with 24-hour urinary excretion and is considered a hallmark of CKD diagnosis and prognostication [2].

Despite placing a strong emphasis on lifestyle management, better control of co-morbidities and the integration of innovative medications in the management of CKD, there remains a high burden of disease globally. The 2012 KDIGO clinical practice guidelines suggested regular screening of urine ACR and estimated glomerular filtration rate (eGFR) was essential for accurately diagnosing CKD and implementing interventions, but stopped short of addressing the key question around population screening for CKD [1]. When determining what a good screening programme is, it is argued it should allow for early detection and effective treatment of diseases, mitigate risks through early identification and modification of risk factors, as well as ensuring the safety, cost-effectiveness and widespread availability of the screening test.

**Figure 1:**
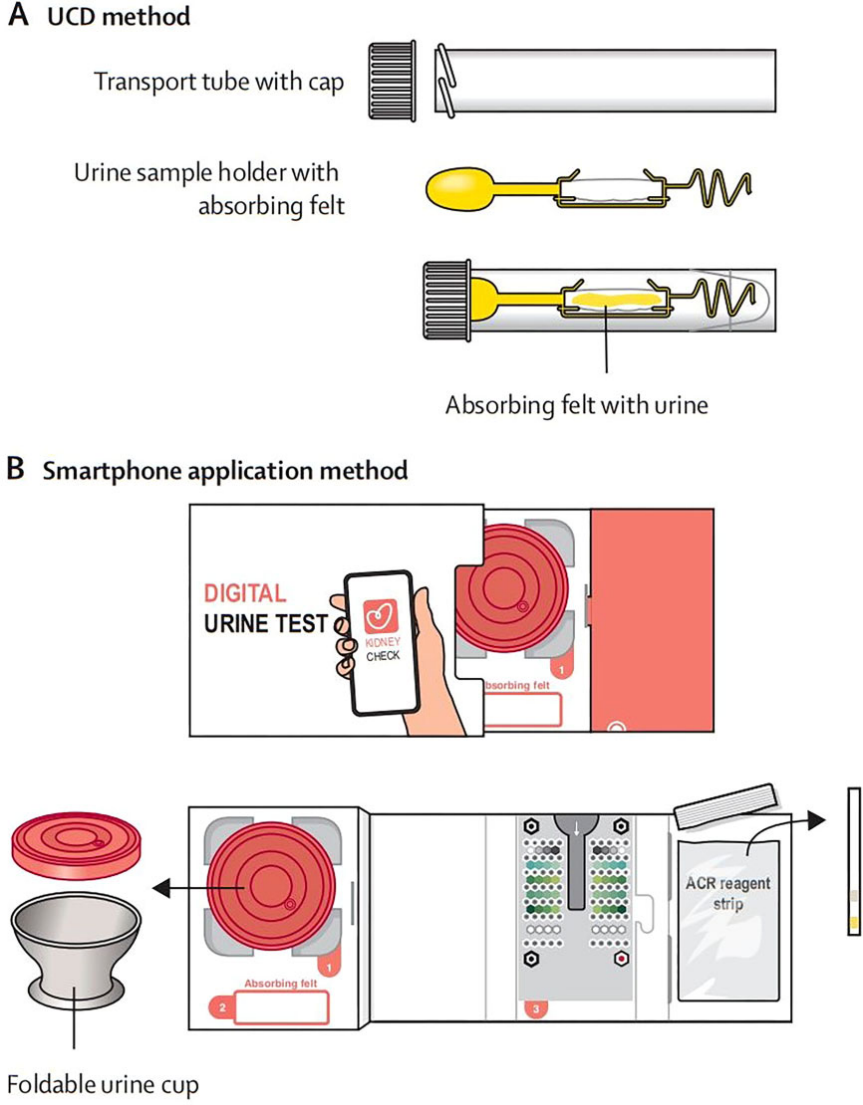
Shows the two home-based screening methods: (**A**) urine collection device (UCD) or (**B**) electronic health method based on a smartphone application, taken from van Mil *et al.* [3].

The recent Towards Home-based Albuminuria Screening (THOMAS) study aimed to evaluate the effectiveness of home-based albuminuria screening methods in the general population [3]. The prospective screening study invited 15 074 Dutch individuals aged 45–80 years to take part in the study between November 2019 and March 2021. Individuals were randomly assigned to one of two home-based screening methods: urine collection device (UCD) or an electronic health method based on a smartphone application (Fig. 1). Positive results were confirmed on a second or third test and participants were then invited for elaborate screening to determine CKD and CVD risk factors.

The participation rate for the home-based albuminuria screening was higher for the UCD method than the smartphone application method (59.4% and 44.3%, respectively) across all strata of sex, age, and socioeconomic status. It was also found to have a higher sensitivity and specificity compared to the smartphone app method. The results of the smartphone app were unreliable due to a significant number of false-positives and too few true-positives, limiting the study's findings. In those with a positive UCD screening result, 96.8% had CKD and or CVD risk factors and 63.7% had at least one newly diagnosed risk factor that may have benefited from intervention.

Who to screen remains unclear as does the overall cost effectiveness (Table 1), as previous population-based studies have shown no significant cost benefit when screening for CKD [4]. In the THOMAS study using the UCD method, the number needed to screen to identify one participant with newly diagnosed increased ACR was 58; however, the number of patients screened who received new treatment was <1%. In previous studies, a selective approach to screen only high-risk patients including those with diabetes, hypertension, familial history, or high-risk ethnic groups has been demonstrated to be cost-effective and carries a higher predictive value [5]. In the THOMAS study 25% had a urine ACR >30 mg/mmol alongside diabetes and hypertension, highlighting that albuminuria in the general population is often a complication rather than cause of other co-morbidities.

**Table 1:** Pros and cons of screening for chronic kidney disease in the general population and in individuals deemed at risk. At-risk individuals include those with diabetes, hypertension, familial history and specific ethnic groups [5].

	**Screening the general population**	**Screening those at risk**
**PROs**	• Quick, non-invasive and acceptable to most individuals • Allows for the management of risk factors and early intervention with effective treatment • Opportunity for patient education • Promotes self-management and shared decision making	• Tailored screening to account for genetic, demographic, and environmental factors • Shown to be more cost-effective • Allows for more targeted and patient-centred treatment • May reduce the progression and therefore burden on KRT services • Encourages an MDT approach to management • Increased observation and follow-up in high-risk individuals
**CONs**	• False positives and false negatives • Not hugely cost-effective • Potential for over diagnosis and over treatment • No long-term data to provide it makes a difference to overall outcomes • Increased health anxiety • Needs to be equitable and allow for equal access • Burden on healthcare resources and primary care services	• Still may not improve overall outcomes • No evidence to suggest an improvement in health-related quality of life outcomes • Screening with eGFR may identify a greater number of at-risk people than screening with urinalysis

CKD: chronic kidney disease; eGFR: estimated glomerular filtration rate ; KRT: kidney replacement therapy; MDT: multidisciplinary team

Over the past decade, newer treatments such as sodium-glucose co-transporter-2 (SGLT2) inhibitors, glucagon-like peptide (GLP1) receptor agonists and selective mineralocorticoid receptor antagonists (MRA), have been demonstrated to improve cardiac and renal outcomes [6], offering potential benefits to those identified from albuminuria screening. However, when comparing screening studies such as the THOMAS study to participants selected for randomised control trials (RCTs) looking at these newer agents, there are significant differences. The median eGFR in THOMAS was 70.9 ± 21.8 ml/min, compared to 43.2 ± 12.3 ml/min in DAPA-CKD, for example [7]. The proportion of participants with CVD, type 2 diabetes mellitus (T2DM) and raised urine ACR measurements was significantly higher in RCTs such as FIDELIO-DKD [8], DAPA-CKD [7], and EMPA-KIDNEY [9] compared to the general population included in the THOMAS study and other population-based screening studies [4]. It remains uncertain as to whether novel treatments would provide any benefit to CKD outcomes within populations characterised by lower rates of CVD, T2DM, and reduced ACRs. Instead, identifying undiagnosed co-morbidities through alternative screening methods may offer a more cost-efficient and simpler approach to determine patients who are most likely to benefit from therapeutic intervention.

Despite the large cohort size in the THOMAS study, uncertainties around population screening remain, including if early identification actually leads to improved long-term outcomes and if the infrastructure and healthcare resources can manage with home-based screening methods on a mass scale. The interval of screening and screening of older adults in whom small amounts of albuminuria is often present and a result of vascular ageing, is another important factor that is seldom reported in the literature but has the potential to impact on the effectiveness of any future screening programme.

We concluded that while population-based screening effectively diagnoses albuminuria, a more impactful and cost-effective approach may involve identifying at-risk populations or screening those who have the most to gain from therapeutic interventions. Further research is needed to determine the longer-term impact of screening on preventing adverse kidney and cardiovascular outcomes.
